# Spatially Filtered Emotional Faces Dominate during Binocular Rivalry

**DOI:** 10.3390/brainsci10120998

**Published:** 2020-12-17

**Authors:** Maria Teresa Turano, Fiorenza Giganti, Gioele Gavazzi, Simone Lamberto, Giorgio Gronchi, Fabio Giovannelli, Andrea Peru, Maria Pia Viggiano

**Affiliations:** 1 Department of Neuroscience, Psychology, Drug Research & Child’s Health, University of Florence, 50100 Florence, Italy; mariateresa.turano@gmail.com (M.T.T.); fiorenza.giganti@unifi.it (F.G.); simonelamberto90@gmail.com (S.L.); giorgio.gronchi@unifi.it (G.G.); fabio.giovannelli@unifi.it (F.G.); andrea.peru@unifi.it (A.P.); 2 Fondazione Turano Onlus, 00195 Roma, Italy; 3 Diagnostic and Nuclear Research Institute, IRCCS SDN, 80121 Napoli, Italy; gioele.gavazzi@unifi.it

**Keywords:** binocular rivalry, emotions, facial expressions, happy bias affect, spatial frequencies

## Abstract

The present investigation explores the role of bottom-up and top-down factors in the recognition of emotional facial expressions during binocular rivalry. We manipulated spatial frequencies (SF) and emotive features and asked subjects to indicate whether the emotional or the neutral expression was dominant during binocular rivalry. Controlling the bottom-up saliency with a computational model, physically comparable happy and fearful faces were presented dichoptically with neutral faces. The results showed the dominance of emotional faces over neutral ones. In particular, happy faces were reported more frequently as the first dominant percept even in the presence of coarse information (at a low SF level: 2–6 cycle/degree). Following current theories of emotion processing, the results provide further support for the influence of positive compared to negative meaning on binocular rivalry and, for the first time, showed that individuals perceive the affective quality of happiness even in the absence of details in the visual display. Furthermore, our findings represent an advance in knowledge regarding the association between the high- and low-level mechanisms behind binocular rivalry.

## 1. Introduction

Emotional facial expressions are the most relevant social cues in everyday human interactions. From an evolutionary perspective, emotions have evolved in order to provide adaptive regulation of our behavior, helping the individual to evaluate the presence of threats or potential mates, and to avoid or approach them depending on whether or not they constitute a relevant concern [1]. Aside from being detected more rapidly in the visual stream, evidence also suggests that emotional facial expressions are more likely to come into awareness and resist failures of attention [2,3]. Several studies have also posited the existence of neural modules with long-standing evolutionary roots, which would be activated preferentially by such stimuli (for a review, see references [4,5]). The amygdala shows a greater fMRI response to fearful and happy faces as compared to neutral faces, even during periods of suppression [6]. Specifically, the interaction between the perigenual prefrontal cortex and amygdala modulates the threshold for awareness of emotional stimuli [7].

Although emotional stimuli (including faces) are recognized quickly, there is no convergence of the results on the neural advantage regarding emotional valence. For example, it has been repeatedly observed that happy faces are recognized more quickly and more accurately than any other facial expression [8]. This is evidence of the fact that positive emotion promotes well-being [9] and, therefore, happy faces are more likely to be chosen during early decisional processes. Conversely, other studies reported that fearful expressions are perceived more quickly than happy [10,11] and neutral ones [12].

Despite our ability to consciously produce and understand facial expressions, in many cases, we obtain emotional information from faces by using pre-conscious processing [13]. This pre-attentive processing results in better stimulus detection [14] and relies on brain mechanisms at least partially dissociable from attentive ones [15].

In pursuit of clues to identify the mechanism of perceptual awareness, binocular rivalry affords unique windows and provides insights into how the visual system handles visual decisional processes. When two different visual stimuli are presented simultaneously to both eyes, they usually do not merge into a single stable combination, but compete for exclusive perceptual dominance. If both stimuli have similar bottom-up salience, such as the same spatial frequency levels, human observers typically experience a perceptual alternation between the two stimuli every few seconds, a phenomenon called binocular rivalry [16]. Whenever one of the two rival stimuli dominates conscious perception, the other respective stimulus is suppressed from conscious awareness [17,18]. However, if one of the two stimuli has a higher top-down salience (e.g., emotional valence) compared to the other, it dominates over time [19,20]. Indeed, both top-down and bottom-up factors, such as high- and low-level properties, can influence the perceptual switching and the duration of dominance periods during binocular rivalry. Earlier studies documented low-level properties, i.e., contrast [21], motion [22], and spatial frequency [23], as the most important forces behind driving which binocular rivalry mechanism is most able to affect the duration of dominance period. Therefore, for some time, researchers believed rivalry was a low-level process concerning only stimulus strengths, such as brightness, contrast, and spatial frequency [24,25]. However, after the pioneering study by Engel [26], the literature began to view rivalry as a high-level process concerning top-down stimuli dimensions, such as their meaning [27,28]. In 2005, van Ee and colleagues [29] claimed that the mechanism behind rivalry (i.e., cycles of perceptual dominance and suppression) is largely independent on voluntary control, engages neural stages along several neural visual pathways, and thus is likely the result of different neural processes [16]. However, even if it has been proposed that binocular rivalry is resolved early in the visual pathway [17], top-down salience could also make a difference.

Given our limited ability to consciously process all the information in our environment (including facial expressions), we can assume that top-down factors, such as the emotional meaning of faces might have priority access to visual awareness during binocular rivalry [19]. How the emotion interacts with sensorial characteristics, such as the composition of visual inputs, in influencing visual decisional processing remains unclear.

In the present study, we aimed to explore the role of bottom-up and top-down factors in the recognition of emotional facial expressions during binocular rivalry. In particular, we manipulated spatial frequencies (SF) and emotive features. Recently, growing interest has arisen concerning low spatial frequencies (LSFs) specificity in the emotional response to happy faces [30,31]; for instance, happy expressions preceded by global processing were identified faster as compared with local processing, and vice versa [32]. In addition, global processing [33,34] has been identified as facilitating identification of faces with a happy expression while local processing facilitated the identification of faces with negative expressions [35]. It is worth noting that the recognition of emotional facial expressions displaying various levels of detail, such as SF, has not been studied previously during binocular rivalry, even if both bottom-up and top-down factors have been shown to trigger rivalry processes. The recent literature has highlighted that the golden standard approach to control the influence of low-level properties of the stimuli is employing inverted stimuli [36,37]. However, we chose to control the role of bottom-up information using the Itti and Koch saliency model [38,39]. This computational model analyzes natural images by simulating the early processing stages of the human visual system (e.g., luminance, color, and orientation) with a feed-forward feature-extraction architecture. The resulting map is able to detect salient objects in complex scenes where locations of higher salience (e.g., salient traffic signs) are more likely to be fixated [40,41]. Several studies [42,43,44,45] have successfully employed this model (and the associated Saliency toolbox) to control the bottom-up saliency of stimuli.

Given the above premises, the present study examined the joint effects of the social relevance of facial stimuli and the role of SF during binocular rivalry, with the aim of investigating which (by keeping contrast and brightness constant) SF-ranges are most relevant in terms of emotional advantage for perceptual dominance during binocular rivalry. For this purpose, happy, fearful, and neutral faces were presented and different bands of SF (very-low, medium-low, low, and broad) were used to examine dominance periods. We selected faces with opposed valence (happy and fearful) that—according to the approach/withdrawal evolutionary model—should inspire opposite reactions [46]. We generated several spatially filtered face stimuli and subsequently created a visual array in which facial expressions were presented dichoptically (neutral and happy or neutral and fearful) during an emotional–binocular rivalry paradigm. We recorded under binocular conditions: (1) the perceptual dominance (i.e., the first dominant percept) between neutral and emotional faces as a function of different bands of SF; and (2) the emotional dominance (i.e., happy and fearful expressions) duration as a function of different bands of SF (very-low, medium-low, low, and broad).

## 2. Materials and Methods

### 2.1. Participants

Eighteen young adults (Mage = 22.7, SDage = 4, nine males) participated in the experiment after giving written informed consent in accordance with the Declaration of Helsinki. Exclusion criteria were a history of neurological or psychiatric diseases, and alcohol or substance abuse. All participants reported normal vision and were right-handed. The study—performed according to the Declaration of Helsinki—is part of a set of behavioral and non-invasive studies on face recognition processing, which were approved by the Research Committee of the University of Florence (protocol number 17245_2012).

### 2.2. Stimuli

We used 96 digitized grayscale frontal view images of male and female individuals selected from the Radboud Faces Database [47], showing neutral (48 images, 24 males), happy (24 images, 12 males), and fearful (24 images, 12 males) facial expressions. We selected the 96 stimuli to be used in our experiment on the basis of the results of a validation study [47]. According to these findings, all the frontal view faces included in the database were perceived as emotional with an overall agreement of 82% (median 88%, SD 19%).

**Figure 1:**
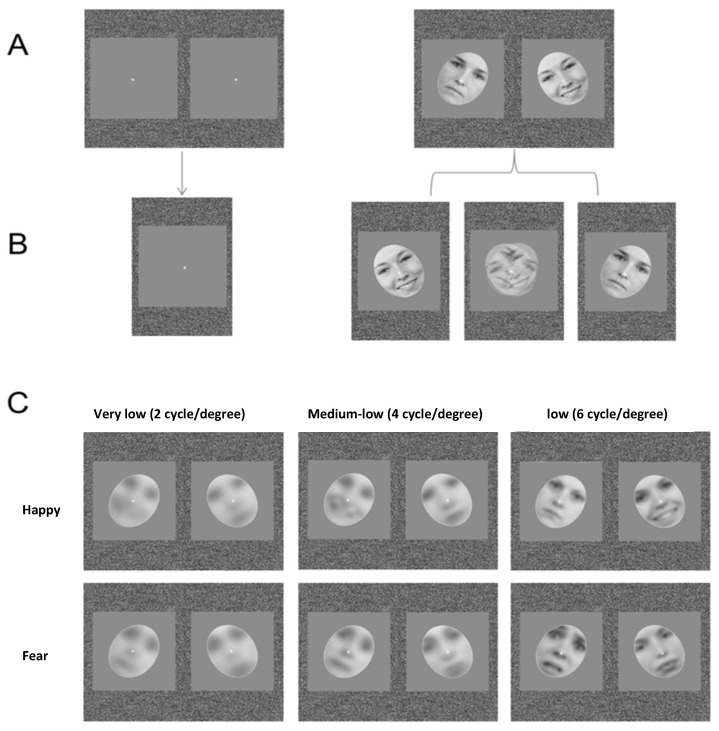
The binocular rivalry paradigm. (**A**) Happy and fearful faces were presented dichoptically along with neutral faces through a mirror stereoscope. (**B**) Participants were asked to report any change in their perception (emotional, neutral, or mixed). (**C**) Examples of filtered happy and fearful faces.

These face stimuli had a size of 1024 × 681 pixels and a resolution of 300 dpi. For the filtering process, we applied the procedure described by Vannucci and colleagues [48]. Firstly, all stimuli were normalized to have the same mean luminance and contrast ranges. Then, 37 face stimuli were filtered using Matlab codes in order to remove specific ranges of spatial frequencies from their spectrum. This filtering process created a multiresolution representation of each image. The different resolutions were obtained by means of a digital filter applied to the bi-dimensional array representing the original image. The multiresolution filter that was selected was the Gaussian mask which performed low-pass filtering (two-dimensional Gaussian convolution). The widths of the filtering windows were the key parameters that determined the bandwidth of the filter. We used three different ascending resolution levels measured on a logarithmic scale of decibels: very-low (2 cycle/degree), medium-low (4 cycle/degree), and low (6 cycle/degree) (see Figure 1). Therefore, we used four different bands in the experiment: one broad and three filtered.

After the filtering procedures, using Adobe Photoshop program, we cropped all faces from hairline to chin and fit them, reducing their size, in a gray “frame” sized 8.89 by 9.16 visual degrees (330 by 340 pixels) with an empty oval window containing the face stimulus sized 6.12 by 5.53 visual degrees (227 by 205 pixels). We created in total 48 visual arrays containing random noise and two spatially aligned framed face stimuli (one neutral and one happy or fearful), which were displayed towards two monocular fixation points (placed at the same position, around the nose of each stimulus). Note that the frames, in which the faces were fitted, were horizontally tilted: the face stimulus presented to the left eye was tilted to the left, and the face stimulus presented to the right eye was tilted to the right (see Figure 1).

Stimuli were tilted in order to present opposing orientations, ensuring the occurrence of perceptual alternation and to induce rivalry [49].

Viewed through the stereoscope, the stimuli included in each visual array presented the same spatial frequencies but different facial expressions: one neutral and the other one happy or fearful.

To rule out the possibility that the visual features of the stimuli (such as the presence of teeth vs. the absence of teeth) could act as confounding variable [50], the saliency model of Itti and Koch [38,39] was employed. The approach we used analyzes natural images by simulating the early processing stages of the human visual system (e.g., luminance, color, and orientation) with a feed-forward feature-extraction architecture. The resulting map is able to detect salient objects in complex scenes where locations of higher salience (e.g., salient traffic signs) are more likely to be fixated [40,41].

Using the SaliencyToolbox 2.3 [51] in default settings, the saliency map of each image was computed. For each corresponding pair of stimuli, we computed the pixelwise difference in the resulting maps via a paired sample *t*-test. Pixels associated with the background that coincided in both images were excluded. The analysis of visual features of stimuli showed that neither of the images in the corresponding pairs differed in a statistically significant way (for all: *p* > 0.1).

In total, each participant was presented with 48 dichoptical trials. In each trial, a neutral face was presented along with an emotional one, 12 for each low-SF band (i.e., very-low, medium-low, low, and broad), so that during each LSF band dichoptical presentation, we presented at total of 12 neutral faces and 12 emotive faces.

### 2.3. Apparatus and Procedures

E-prime software and E-basic language codes were programmed to run the experiment and collect the data. All stimuli were presented on a PC monitor (1024 × 768 h) with a 60 Hz refresh rate. Each of the 48 visual arrays containing the face pairs was presented once in random order for 30 s. For all instructions, we projected identical material to both eyes, so as to create normal vision.

Responses were recorded using a response-box with three horizontally placed keys (emotional, neutral, and mixed perception). A chin rest ensured a viewing distance of 57 cm. While viewing the rival stimuli through a mirror stereoscope [52], participants concurrently reported what they perceived using the response-box. The order of the response-box buttons was counterbalanced across participants. Participants were asked to report any change in their perception as quickly as possible by pressing response buttons using the index finger of their dominant hand. To avoid coding errors that are often observed when specific emotions are categorized [53,54], we simply instructed participants to indicate whether the emotional or the neutral expression was dominant. If they saw a mixture or if none of the two incongruent pictures clearly appeared in the foreground, they reported “mixed”, otherwise, they reported “neutral” or “emotional”. Before starting the task, participants performed two independent trials to familiarize themselves with the procedure. At the beginning of each rival presentation, participants were asked to report the first dominant perception only when one of the rival face stimuli was perceived as exclusive. After the first dominant percept (following the first keypress), they were also required to report the transition from one dominant image to the other (i.e., those periods in which they did not clearly perceive a dominant stimulus but a mix of monocular stimuli). Between the stimuli presentations, there was a pause of 5 s, consisting of two gray frames on a white noise background. Fixation points were always displayed.

### 2.4. Statistical Analyses

For each participant and across each trial, we collected the first dominant perception and the emotional dominance duration.

The first dominant perception (FDP) measure was calculated for each expression and for each SF band as the average relative frequency of trials in which participants reported the emotive compared to the neutral expression as the first perception. To prevent coding errors of expressions during this type of task [53], participants were only required to indicate whether the dominant stimulus was neutral or emotive, therefore participants did not directly report which specific facial expression (i.e., happy or fearful) was dominant. We derived the frequencies for happy and fearful emotions for each SF band based on the type of trial presented to the participant.

Following Levelt’s approach [25], for each participant and for each SF band (very-low, medium-low, low, and broad), we calculated the emotive dominance duration (EDD) for each facial expression by means of the following formula:

emotive dominance duration = ED/(ED + EN)

where ED represents the cumulative duration of the happy or fearful dominant perception and EN indicates the cumulative duration of the dominant neutral percept. It is important to note that in determining EDD, we used a trial-by-trial approach so that the effect of the duration of periods of mixed perception was not considered.

Preliminarily, we checked if data were normally distributed using the Kolmogorov–Smirnov test. In order to compare emotive and neutral faces, for both FDP and EDD measures and for each SF band, we compared the mean value to the reference value of 50% by means of a one-sample Student’s *t* test.

Differences between trials in which happy faces were compared to neutral faces and trials in which fearful faces were compared to neutral faces were assessed by means of a repeated measures analysis of variance (ANOVA 2 × 4) for FDP and EDD separately. In both cases, the factors taken into account were expressions (two levels: happy and fearful) and spatial frequencies (four levels: very-low, medium-low, low, and broad). Degrees of freedom for repeated measure effects were Greenhouse–Geisser corrected. Post-hoc comparisons were Bonferroni corrected.

## 3. Results

### 3.1. First Dominant Percept

**Figure 2:**
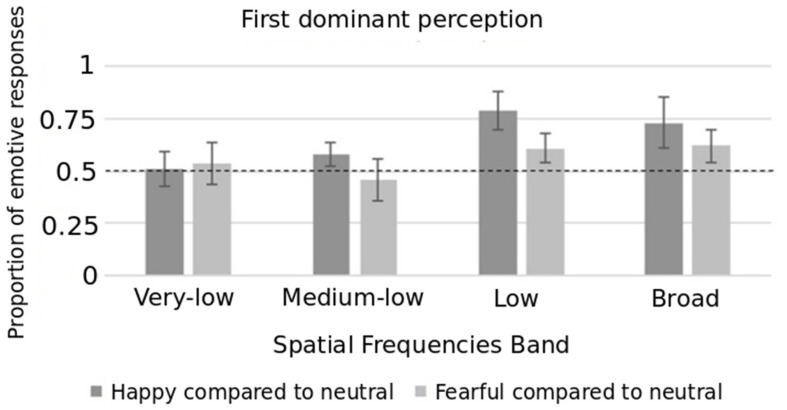
Average frequency of first dominant perception as function of spatial frequency bands with 95% confidence interval. The dotted line represents the reference proportion of 50%.

Both indices were normally distributed according to the Kolmogorov–Smirnov test (for all: *p* > 0.070). Figure 2 reports FDP mean values across the happy–neutral and fearful–neutral conditions for each SF band (very-low, medium-low, low, broad). Happy expressions showed an advantage compared to neutral expression in terms of FDP for all SFs with the exception of very-low SF (*t*(17) = 0.30, *p* = 0.769). Indeed, the FDP value associated to happy faces was significantly higher than 50% during medium-low (*t*(17) = 2.40, *p* = 0.028), low (*t*(17) = 6.68, *p* < 0.001), and broad (*t*(17) = 3.62, *p* = 0.002) conditions. With regard to the fearful–neutral comparison, the advantage of fearful expression was observed only within low (*t*(17) = 2.26, *p* = 0.038) and broad (*t*(17) = 2.78, *p* = 0.013) SFs, but it was not present within very-low (*t*(17) = 0.80, *p* = 0.432) and medium-low (*t*(17) = −0.91, *p* = 0.374) SF stimuli.

The repeated measure ANOVA revealed the main effect of expressions (F(1, 17) = 14.76, *p* = 0.001, η_p_^2^ = 0.465). Participants reported more frequently as first perception the facial expression of happiness in the happy/neutral trials compared to fearful expression in the fearful/neutral trials. The main effect of SFs was also statistically significant (F(3, 51) = 8.55, *p* = 0.001, η_p_^2^ = 0.335). Participants reported more frequently as first perception an emotive face (independently from the specific expression) in low SF than in very-low (*p* = 0.004) and in medium-low SF (*p* < 0.001).

These effects were further qualified by the significant interaction between expressions and SFs (F(3, 51) = 2.81, *p* = 0.049, η_p_^2^ = 0.142). The FDP value was higher for the happy trials than the fearful trials within the broad SF band (*t*(17) = 2.13, *p* = 0.049), low SF band (*t*(17) = 3.29, *p* = 0.004), and medium-low SF band (*t*(17) = 2.32, *p* = 0.033), but not within the very-low SF band (*t*(17) = −0.53, *p* = 0.603).

### 3.2. Emotive Dominance Duration

**Figure 3:**
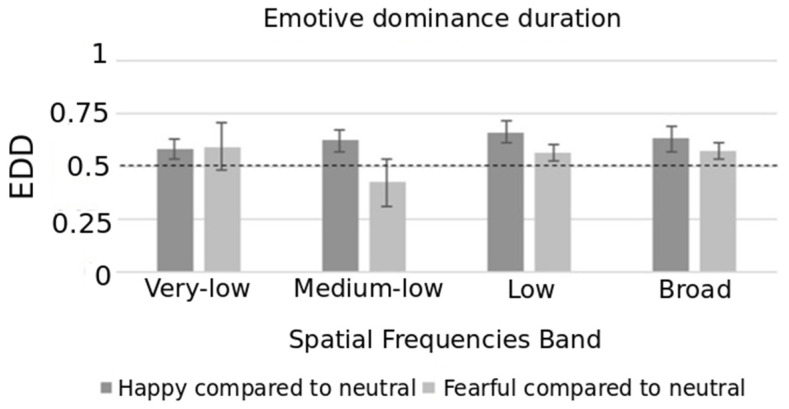
Average emotive dominance duration as a function of spatial frequency bands with 95% confidence interval. The dotted line represents the reference proportion of 50%.

EDD mean values measured in the happy–neutral and fearful–neutral trials as a function of each SF band (very-low, medium-low, low, broad) are reported in Figure 3. Happy expressions showed an advantage compared to neutral expression in terms of EDD for all SFs: the mean values associated to happy faces were significantly higher than 50% during very-low (*t*(17) = 2.87, *p* = 0.011), medium-low (*t*(17) = 3.47, *p* = 0.003), low (*t*(17) = 6.07, *p* < 0.001) and broad (*t*(17) = 4.00, *p* = 0.001) conditions. In the fearful–neutral trials, the advantage of fearful expression was observed only within low (*t*(17) = 2.23, *p* = 0.040) and broad (*t*(17) = 2.90, *p* = 0.010) SFs. No differences were observed with the very-low (*t*(17) = 1.94, *p* = 0.069) and medium-low (*t*(17) = −1.93, *p* = 0.070) SF stimuli.

The repeated measure ANOVA revealed the main effect of expressions (F(1, 17) = 15.80, *p* = 0.001, η_p_^2^ = 0.482) and of SFs (F(3, 51) = 3.83, *p* = 0.037, η_p_^2^ = 0.184). The dominance duration of happiness in the happy–neutral trials was higher compared to fearful expression in the fearful–neutral trials. EDD mean values (independently from the specific expression) observed during low were higher than the values observed during medium-low SF stimuli (*p* < 0.001). The interaction effect was also significant (F(3, 51) = 3.20, *p* = 0.031, η_p_^2^ = 0.158). The EDD value was higher for the happy trials than the fearful trials within the broad SF band (*t*(17) = 2.15, *p* = 0.046), low SF band (*t*(17) = 3.02, *p* = 0.008), medium-low SF band (*t*(17) = 3.71, *p* = 0.002), but not within the very-low SF band (*t*(17) = −0.05, *p* = 0.958).

## 4. Discussion

The present study was designed to investigate both the bottom-up effects of spatial frequency manipulations and the top-down effects of emotional content on the perception of faces during binocular rivalry. Namely, spatially filtered and unfiltered happy and fearful faces, both of them particularly salient to human vision, were presented dichoptically along with neutral faces. Results provide evidence of an emotional bias that is more pronounced for happy faces (the happy face advantage).

We observed an “emotive advantage”: emotional faces (happy and fearful) were better detected (as measured by first perception) than neutral faces despite being filtered at increasing levels of SF. In particular, the happy over neutral face advantage was already observed from medium-low SF levels, whereas the fearful over neutral face advantage was only observed from low-level SF levels. Therefore, we confirmed the previous finding that a more meaningful stimulus (i.e., an emotional face) has perceptual predominance over a less meaningful stimulus (i.e., a neutral face) [19]. The first dominant percept is considered as an index of the perceptual strength of facial expression during binocular rivalry, which is independent of habituation or inaccuracy over time. This suggests that the visual system is sensitive to stimuli that signal emotional information and our data are consistent with theories demonstrating the detection and categorization of facial expressions (emotional vs. neutral) as being performed at a very early processing stage [54]. The data herein demonstrated, in the presence of coarse information, a prioritization of happy faces over the neutral ones—with respect to fearful faces—in the competition for awareness of emotional valence of stimulus emerged. Such observations support the notion that coarse, rapid, magnocellular input to the brain is sufficient for the evaluation and subsequent detection of emotional stimuli. Our results are consistent with the hypothesis of distinct processing for happiness/positive as compared to fear/negative, i.e., the happy bias effect [55]. This effect emerged even when a coarse representation of stimuli was presented, and this was not due to distinctive facial features of the smiling faces such as the salient marker of a mouth showing teeth. Indeed, we gradually reduced the amount of spatial information and only when a large filter was applied (amplitude of 46 db), vastly degrading the stimuli, happiness and fear were perceived as dominant with the same frequency. The band of very-low SF used in this study could therefore be considered as a post-hoc putative control condition.

The ability to select is crucial when our brain needs to evaluate internal or external stimuli and direct its early attention. These automatic processes are termed as “silent” since they occur outside conscious awareness, and are related to detection processes, analysis, and identification of stimuli [56]. We evaluated this sensorial gating effectively with binocular rivalry. The emotional valence of happy faces, particularly relevant to human vision, is probably evaluated without awareness [13], and preferentially proposed to conscious perception. This happy advantage could be related to the existence of a specialized and innate mechanism that promotes positive stimuli to awareness, as well as to a learned mechanism that improves our sensory processing of positive stimuli that cause pleasure and well-being [9]. For example, increased positive emotion promotes creative thinking, social connection with others [57], emotional resilience in the face of stressors [58], and better physical health [59]. Although some authors have embraced the hypothesis of a specific competence for happy expression that triggers the happy bias effect [60], the existence of an efficient attentional mechanism with an important adaptive function cannot be excluded.

The present data could be framed within theories that posit an emotive processing gating [61], which interrupts the incoming negative information and promotes the positive ones which will be processed in the subsequent perceptual and recognition stages. Thus, it could be the case that preconscious visual processes selectively promote happy faces that resemble conspecific stimuli to conscious perception, presumably because of their social relevance. However, since the top-down effects found in binocular rivalry (and similar techniques) have been attributed to perceptual properties [62,63,64], we ruled out the potential confounding variable related to the bottom-up saliency with the saliency map model of Itti and Koch [38,39], as was done in previous research [42,43,44,45]. Consistently, Barrneman et al. [65] also found a general top-down effect of emotional expression in face perception in a binocular rivalry paradigm excluding the influence of low-level properties on the basis of a control experiment with inverted faces.

However, although our results suggest a top-down effect, we cannot exclude with certainty that the expression recognition performance is affected by the current spatial filters and this effect could be related to the magnitude of the expression effects. Therefore, as a limitation, we are aware that a control of the magnitude spectra of images would have been necessary to ascertain whether the magnitude spectra were suffice for decoding the emotional expression. It would be crucial to understand whether the ‘bottom-up saliency’ remains equal when visual sensitivity can still differ between the conditions, even if no local saliency differences are found. Future studies should address this issue by taking the average magnitude spectra of the image in one filtering condition and use those to recreate the individual images with an inverse Fast Fourier Transform where the unique phase spectra are applied to the average magnitude spectra. According to our expectations, an experiment with the application of this technique to control for the images’ spatial frequencies should replicate the same effects we observed.

Apart from our results limitations, this study is an important step in opening interesting avenues for future research. For example, whether amygdala activity to presented threat stimuli (in response to either low-level or affective properties) has a functional role in modulations, promoting or stopping their detection, remains an interesting question. It is important to note that our results were ultimately based on keypresses (i.e., participants self-reported their precepts) and, thus, we cannot fully eliminate the possibility of biased self-reporting.

Future studies should incorporate control conditions (e.g., using ERPs) to replicate and corroborate the present findings. To this end, we have recently investigated the electrophysiological coding of all the basic facial expressions plus neutral ones using a repetition suppression paradigm to assess emotion modulations on the early N170 face-sensitive ERP component [66]. While we observed occipito-temporal responses for fear on the N170 time window, other researchers have also shown greater N170 modulations for other facial expressions, such as anger and happiness (for a review, see references [67,68,69]). Differences in the experimental designs have been proposed to explain different results [66]. Indeed, the advantage for processing happy faces has been mostly demonstrated in long-term memory tasks [69]. Experiments in the context of binocular rivalry could help clarify this debate.

## 5. Conclusions

The main finding of this study is that emotional meaning modulates binocular rivalry: when emotional and neutral monocular faces were presented dichotoptically, emotional faces, and in particular happy faces, were detected more frequently than neutral ones. Therefore, we support the view that emotion routinely alters our perception across many levels of visual perception and from the very early stages concerning decisional processes. Importantly, our data suggest a happy advantage, which persists even when low-level image properties, a driving force behind binocular rivalry, were manipulated. Keeping the contrast constant, even when we limited the spatial frequency range, the duration of perceptual predominance of happy faces did not decrease. One important explanation for this finding may be that it is vital for an organism to attend to information that is of high importance for behavioral goals, because it will assist in guiding both actions and thoughts [70,71]. Thus, emotional facial expressions, which are known to convey a high adaptive value in signaling crucial social information, undergo preferential perceptual processing [70,72,73]. Experiments of the neural correlate of consciousness in the context of binocular rivalry could help clarify the debate as to whether positive and negative content modulates the conscious perception differently [74].
